# Association between cardiovascular health and markers of liver function: a cross-sectional study from NHANES 2005–2018

**DOI:** 10.3389/fmed.2025.1538654

**Published:** 2025-03-12

**Authors:** Huang Yu, Tingyi Zhang, Yankun Liu, Wang Wang, Ziyi Guan, Ping Li

**Affiliations:** Department of Cardiovascular Medicine, The Second Affiliated Hospital, Jiangxi Medical College, Nanchang University, Nanchang, Jiangxi, China

**Keywords:** CVH, liver function, NHANES, cross-sectional study, relationship

## Abstract

**Background:**

Cardiovascular health (CVH) has been associated with various systemic diseases. However, the relationship between CVH, as measured by Life’s Essential 8 (LE8), and liver function markers in the general population remains poorly understood.

**Methods:**

This study analyzed data from 21,156 participants (aged ≥ 20) from the NHANES 2005–2018 to investigate the associations between CVH and liver function markers [alanine aminotransferase (ALT), aspartate aminotransferase (AST), gamma-glutamyl transferase (GGT), alkaline phosphatase (ALP), albumin and AST/ALT ratio]. Linear regression models were used, along with a restricted cubic spline (RCS) to assess dose-response. Weighted quantile sum (WQS) regression and quantile g-computation (QGC) analyses were employed to evaluate the association between CVH and liver function markers.

**Results:**

Linear regression analysis showed that each 1-point increase in CVH score was significantly associated with decreased levels of liver enzymes [ALT: −0.200 U/L (95% CI: −0.223, −0.176), AST: −0.043 U/L (−0.062, −0.024), GGT: −0.453 U/L (−0.509, −0.397), ALP: −0.310 U/L (−0.340, −0.281)] and increased levels of albumin [0.040 g/dL (0.036, 0.045)] and AST/ALT ratio [0.0056 (0.0051, 0.0061)]. Notably, CVH score demonstrated non-linear dose-response relationships with ALT, ALP, and AST/ALT ratio. Age significantly modified these associations, while nicotine exposure, BMI, and blood lipids were identified as primary contributors through WQS and QGC analyses. E-value analysis suggested robustness to unmeasured confounding.

**Conclusion:**

This study demonstrates robust associations between CVH and liver function markers in United States adults, with nicotine exposure, BMI, and blood lipids identified as significant contributors. These findings suggest that maintaining optimal cardiovascular health may have beneficial effects on liver function, highlighting potential targets for integrated prevention strategies.

## 1 Introduction

The liver, as the largest internal organ, plays a significant role in regulating numerous physiological processes, including metabolism, detoxification, and homeostatic regulation (1). Liver diseases account for approximately two million deaths annually, constituting 4% of global mortality (2). Despite the current therapeutic interventions, which primarily comprise liver transplantation and cellular therapy, these approaches face significant limitations. This is due to a critical shortage of donor organs, which results in global transplantation capacity meeting less than 10% of clinical demand (2). Moreover, the occurrence of post-transplantation complications, including immune rejection and impaired long-term outcomes (3), highlights the pressing necessity for the development of more efficacious preventive strategies. The pathogenesis of liver disease involves complex tissue alterations induced by diverse etiological factors, including viral infections, alcohol consumption, pharmaceutical agents, inflammatory processes and metabolic dysfunction (4, 5).

Emerging evidence suggests a crucial bidirectional relationship between cardiovascular and liver health. Non-alcoholic fatty liver disease (NAFLD) is associated with various metabolic syndromes, including obesity, diabetes, and dyslipidemia, which are common risk factors for cardiovascular diseases. Studies have shown that individuals with NAFLD have a higher prevalence of cardiovascular diseases, with some research indicating that NAFLD may independently increase the risk of multi-vessel coronary artery disease and other cardiovascular events (6–8). A systematic review highlighted that NAFLD is linked to a substantial increase in the risk of major cardiovascular events, emphasizing its role as a predictor of cardiovascular morbidity and mortality (8). Additionally, the presence of severe liver fibrosis in NAFLD patients significantly heightens the risk of cardiovascular complications, with some studies reporting a 69% increase in overall mortality due to cardiovascular causes in such patients (9). Conversely, cardiovascular health significantly influences liver function. A prospective cohort study of 3,424 middle-aged and elderly Chinese adults demonstrated that individuals with 5–6 ideal cardiovascular health metrics exhibited a 66% reduction in NAFLD incidence compared to those with 0–2 metrics (10). Supporting this, the Chilean National Health Survey revealed that adults meeting 5–7 ideal cardiovascular health criteria showed 73%, 72%, and 95% lower odds of elevated γ-GT, ALT, and FLI, respectively (11). This bidirectional relationship extends to severe conditions, where cirrhosis patients often develop cardiac complications including diastolic dysfunction and cardiomyopathy (12), while cardiovascular disease can exacerbate liver conditions, particularly in patients undergoing procedures such as liver transplantation, where postoperative cardiovascular events are a leading cause of morbidity and mortality (13).

The American Heart Association’s Life’s Essential 8 (LE8) assessment system provides a comprehensive framework for evaluating cardiovascular health (CVH) through eight indicators: body mass index (BMI), blood glucose, blood pressure, cholesterol, physical activity, diet, nicotine exposure, and sleep duration (14). While individual components of cardiovascular health have been studied in relation to liver function (15–20), these studies have several notable limitations. First, most existing research has focused on isolated cardiovascular risk factors, failing to capture their collective impact on key liver function markers (ALT, AST, GGT, ALP, albumin and AST/ALT ratio). Second, comprehensive analyses utilizing the standardized LE8 metrics to evaluate multiple liver function markers are lacking in current literature, despite LE8 being a validated and comprehensive assessment tool for cardiovascular health. Third, the relative contributions of different CVH components to liver function markers remain poorly characterized.

To address this research gap, we analyzed the relationship between CVH scores measures by LE8 and liver function markers in United States adults using National Health and Nutrition Examination Survey (NHANES) data. Quantile G-computation (QGC) analyses and Weighted Quantile Sum (WQS) regression were applied to evaluate the combined effects of CVH components on liver function in United States adults. Our findings provide crucial insights into the cardiovascular-liver health relationship, supporting evidence-based clinical prevention strategies and informing targeted interventions for liver disease prevention through cardiovascular health management.

## 2 Materials and methods

### 2.1 Data source

The NHANES provided the source data for this investigation. NHANES employs a complex, stratified, multistage probability sampling methodology to generate a nationally representative sample of the non-institutionalized civilian population in the United States. Under the auspices of the National Center for Health Statistics (NCHS), NHANES implements a comprehensive data collection protocol encompassing structured participant interviews, standardized physical examinations, and laboratory assessments. The study protocols received approval from the NCHS Research Ethics Review Board (Protocol #2005-06 and Protocol #2011-17, both with continuations). Written informed consent was obtained from all participants before study enrollment.

### 2.2 Study population

The study population was derived from seven consecutive NHANES cycles (2005–2018), comprising an initial cohort of 70,190 participants. We implemented a systematic exclusion process to establish our final analytical cohort. First, we excluded participants younger than 20 years (*n* = 30,442) and pregnant women (*n* = 711) to minimize the influence of age-related and pregnancy-induced variations in liver function.

To eliminate the confounding effects of viral hepatitis, we excluded participants with serological evidence of viral hepatitis infection (*n* = 3,064). Specifically, individuals who tested positive for hepatitis B core antibody (anti-HBc) using the VITROS Anti-HBc assay system were excluded (*n* = 2,704). For hepatitis C virus (HCV) infection, participants were excluded if they had either confirmed positive results from the Recombinant Immunoblot Assay (RIBA) (*n* = 292) or indeterminate RIBA results combined with positive HCV-RNA findings (*n* = 68).

**FIGURE 1:**
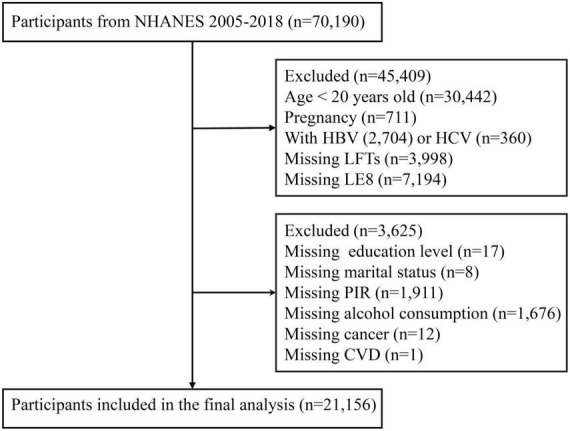
Flowchart of the sample selection from National Health and Nutrition Examination Survey (NHANES) 2005–2018.

Furthermore, we excluded participants lacking hepatic function markers (*n* = 3,998), those with insufficient data for LE8 score calculation (*n* = 7,194) and individuals with incomplete covariate information (*n* = 3,625). The final analytical cohort comprised 21,156 participants (Figure 1).

### 2.3 Markers of liver function

In the NHANES, fasting blood samples were collected from participants at mobile examination centers following standardized protocols. Serum specimens were maintained at 2–8°C until transport to the Collaborative Laboratory Services (Ottumwa, Iowa) for biochemical analysis using a Beckman Coulter DxC800 analyzer. The analytical panel comprised three categories of hepatic biomarkers: aminotransferases (ALT and AST), canalicular membrane enzymes (GGT and ALP), and synthetic proteins (albumin). ALT demonstrates great hepatic specificity, whereas AST exhibits broad tissue distribution across hepatic, cardiac, and skeletal muscle tissues (21). During hepatocellular injury, these aminotransferases are released into circulation, with their serum levels serving as quantitative indicators of liver damage (22). ALP and GGT, localized to the hepatocyte canalicular membrane, show concurrent elevation during cholestatic conditions, facilitating assessment of biliary dysfunction (23). Although ALP is expressed in multiple tissues including bone, kidney, and placenta, its elevation in conjunction with GGT specifically indicates hepatobiliary dysfunction (24). Albumin, synthesized exclusively by hepatocytes, functions as a direct marker of hepatic synthetic capacity, with serum concentrations decreasing markedly during severe hepatic impairment (25). Additionally, the AST/ALT ratio was calculated from serum aminotransferase levels as an additional parameter for evaluating hepatic dysfunction and fibrosis progression (26, 27). This cross-sectional analysis utilized these serum biomarkers to evaluate hepatic function.

### 2.4 Evaluation of CVH

Cardiovascular health was evaluated using the LE8 scoring system, which comprises eight components across two distinct categories (14). The health factors category encompasses physiological parameters: blood glucose, blood pressure, BMI, and non-high-density lipoprotein cholesterol. The health behaviors category includes dietary quality [quantified using the Healthy Eating Index-2015 (HEI-2015) derived from 24 h dietary recalls], physical activity levels, nicotine exposure, and sleep duration. Each component was standardized on a 0–100 scale, with the composite score calculated as the arithmetic mean of all components (detailed methodology provided in Supplementary Table 1). LE8 was stratified into three categories: high (80–100 points), moderate (50–79 points), and low (0–49 points) (14).

### 2.5 Covariates

Covariates encompassed demographic, socioeconomic, clinical, and pharmacological parameters. Demographic variables included age, gender (male/female), and race/ethnicity (categorized as Mexican American, non-Hispanic Black, non-Hispanic White, other Hispanic, and other race/multiracial). Socioeconomic indicators comprised education level (less than high school, high school graduate, college or above), poverty-to-income ratio (PIR; stratified as < 1.3, 1.3–3.5, and ≥ 3.5), and marital status (married/cohabiting, divorced/separated/widowed, never married). Clinical covariates included cardiovascular disease (CVD), chronic kidney disease (CKD), cancer status, and alcohol consumption (defined as ≥ 12 alcoholic beverages annually). Medication-related covariates included both hepatotoxic and hepatoprotective agents.

Cardiovascular disease was defined by physician-diagnosed conditions including congestive heart failure, coronary heart disease, angina pectoris, myocardial infarction, or cerebrovascular accident. CKD was characterized by either an estimated glomerular filtration rate (eGFR) < 60 mL/min/1.73 m^2^ or urine albumin-to-creatinine ratio (ACR) ≥ 30 mg/g, calculated using the Chronic Kidney Disease Epidemiology Collaboration (CKD-EPI) creatinine equation (28). Cancer status was ascertained through self-reported physician diagnoses. Detailed methodological protocols are accessible through the National Health and Nutrition Examination Survey (NHANES) documentation portal.

Medications were stratified into hepatotoxic and hepatoprotective categories. The hepatotoxic classification comprised seven classes: (1) analgesics/antipyretics (paracetamol and NSAIDs), (2) antineoplastic/immunomodulatory agents (exemplified by methotrexate), (3) antimicrobials (amoxicillin-clavulanate, antitubercular agents), (4) antifungals (fluconazole, ketoconazole), (5) lipid-modifying agents (primarily HMG-CoA reductase inhibitors), (6) anticonvulsants (valproic acid, phenytoin, carbamazepine), and (7) miscellaneous hepatotoxic agents (allopurinol, amiodarone).

The hepatoprotective classification encompassed six categories: (1) botanical preparations [Silybum marianum (silymarin), Glycyrrhiza glabra (glycyrrhizin), Schisandra chinensis], (2) micronutrients (α-tocopherol, ascorbic acid, B-complex vitamins, selenium), (3) cellular protectants (reduced glutathione, N-acetylcysteine), (4) established hepatoprotective agents (ursodeoxycholic acid, phosphatidylcholine), (5) probiotic microorganisms (Lactobacillus spp., Bifidobacterium spp.), and (6) proprietary hepatoprotective formulations (Essentiale^®^, Liv.52^®^). The medication identification protocol incorporated both international non-proprietary names and registered trade names.

### 2.6 Statistical analysis

Statistical analyses incorporated complex multi-stage survey design methodologies following NHANES analytical guidelines, with appropriate survey weights (MEC2yr) applied. Continuous variables are presented as survey-weighted means with 95% confidence intervals (CIs), while categorical variables are expressed as survey-weighted percentages with 95% CIs. Between-group comparisons were conducted using weighted variance tests for continuous variables and weighted chi-squared tests for categorical variables. Study participants were stratified into tertiles based on CVH scores, with the lowest tertile serving as the reference group.

The associations between CVH scores and hepatic function markers were examined using multiple linear regression models. Potential non-linear relationships were investigated using restricted cubic spline (RCS) analyses. To evaluate the robustness of the primary findings, stratified analyses were conducted across demographic and socioeconomic subgroups [age, gender, race/ethnicity, educational level, marital status, and poverty-to-income ratio (PIR)], with interaction terms evaluated to assess potential effect modification.

Associations between individual CVH components and hepatic function markers were evaluated using Pearson correlation coefficients. The overall relationship between CVH and liver function markers was analyzed using Weighted Quantile Sum (WQS) regression and quantile g-computation analysis (QGC). WQS regression quantified the cumulative effects of CVH components by constructing a weighted index, where weights were constrained between 0 and 1, with their sum equal to 1. This approach identified the relative contribution of each component to the unidirectional cumulative effect. QGC analysis was employed to examine the directional contribution of individual components to the overall association. In QGC, component weights could be either positive or negative, with positive and negative weights each summing to 1, thereby capturing both direction and magnitude of each component’s contribution. These complementary methods provided distinct insights: WQS regression quantified the cumulative impact of CVH components, while QGC analysis elucidated their contributions through directional effects and relative importance.

To assess potential selection bias, we compared characteristics between included participants (*n* = 21,156) and those excluded due to missing data (*n* = 14,817). We examined differences in demographic characteristics, socioeconomic status, clinical features, and medication use patterns. Standardized differences were calculated to quantify the magnitude of differences between groups, with values < 0.1 indicating negligible differences. Chi-square tests for categorical variables and *t*-tests for continuous variables were performed to assess statistical significance. Unweighted data were used in this sensitivity analysis to directly assess differences between groups. Second, we explored the potential for unmeasured confounding between CVH scores and hepatic function markers by calculating E-values (29). The E-value quantifies the minimum strength of association that an unmeasured confounder would need to have with both the exposure and outcome to explain away the observed association.

Analyses were conducted using R version 4.4.1 (R Foundation for Statistical Computing, Vienna, Austria) and Empower Stats software^1^. Statistical significance was established at a two-sided *P*-value < 0.05.

## 3 Results

### 3.1 Baseline characteristics

The cross-sectional analysis included 21,156 participants stratified by CVH status into low (*n* = 2,687), moderate (*n* = 14,253), and high (*n* = 4,216) categories. The study population consisted of 48.26% males, with a mean age of 47.42 years. Significant differences in demographic and clinical characteristics were observed across CVH categories (all *P* < 0.001). Compared with the low CVH group, participants with high CVH were younger, predominantly Non-Hispanic White, demonstrated higher educational levels and income levels, and exhibited lower prevalence of comorbidities including CKD, cancer, and CVD. Notably, all hepatic function markers showed significant differences across CVH categories (all *P* < 0.001), with a consistent gradient observed from low to high CVH groups (refer to Table 1).

**TABLE 1:** Weighted baseline characteristics of study population.

Characteristics	Overall	Low CVH	Moderate CVH	High CVH	*P*-value
*N*	21,156	2,687	14,253	4,216	**–**
Male, %	48.26	46.40	51.21	40.79	**< 0.0001**
Age, y	47.42 ± 0.27	54.02 ± 0.39	48.50 ± 0.28	41.55 ± 0.43	**< 0.0001**
Race/ethnicity, %					**< 0.0001**
Mexican American	7.69	6.90	8.04	7.03	**–**
Other Hispanic	4.71	3.94	4.74	4.96	**–**
Non-Hispanic White	72.84	69.97	72.43	75.24	**–**
Non-Hispanic Black	9.11	14.39	9.67	5.27	**–**
Other race	5.65	4.80	5.12	7.49	**–**
Education level, %					**< 0.0001**
< High school	13.30	24.92	14.04	6.28	**–**
High school	22.74	31.09	25.24	12.16	**–**
> High school	63.96	43.98	60.72	81.56	**–**
Marital status, %					**< 0.0001**
Married/living with a partner	65.36	59.43	65.98	66.16	**–**
Divorced/separated/widowed	17.63	28.03	18.79	9.95	**–**
Never married	17.01	12.54	15.23	23.89	**–**
PIR, %					**< 0.0001**
< 1.3	18.55	30.39	18.61	13.32	**–**
1.3–3.5	35.61	41.76	36.83	29.58	**–**
≥ 3.5	45.84	27.85	44.56	57.10	**–**
Alcohol consumption, %	76.65	72.32	76.63	78.56	**0.0005**
CKD, %	13.75	29.63	13.64	7.25	**< 0.0001**
Cancer, %	10.25	12.05	10.97	7.46	**< 0.0001**
CVD, %	8.11	20.70	8.19	2.50	**< 0.0001**
Hepatotoxic medications, %	20.11	34.77	21.26	10.62	**< 0.0001**
Hepatoprotective medications, %	1.54	3.15	1.50	0.94	**< 0.0001**
ALT, U/L	24.85 ± 0.14	27.58 ± 0.49	25.72 ± 0.18	21.24 ± 0.22	**< 0.0001**
AST, U/L	24.89 ± 0.12	25.71 ± 0.34	25.12 ± 0.14	23.88 ± 0.23	**< 0.0001**
GGT, U/L	27.17 ± 0.31	37.89 ± 1.25	28.48 ± 0.43	18.88 ± 0.42	**< 0.0001**
ALP, U/L	67.44 ± 0.28	76.27 ± 0.72	68.72 ± 0.33	60.10 ± 0.37	**< 0.0001**
Albumin, g/L	42.78 ± 0.05	41.42 ± 0.10	42.68 ± 0.05	43.63 ± 0.07	**< 0.0001**
AST/ALT	1.11 ± 0.00	1.05 ± 0.01	1.09 ± 0.00	1.21 ± 0.01	**< 0.0001**
CVH score	68.58 ± 0.25	42.18 ± 0.18	66.11 ± 0.12	86.79 ± 0.12	**< 0.0001**
HEI-2015 diet score	39.29 ± 0.47	20.16 ± 0.55	35.05 ± 0.47	59.35 ± 0.66	**< 0.0001**
Physical activity score	72.37 ± 0.50	28.06 ± 1.24	71.45 ± 0.54	93.93 ± 0.38	**< 0.0001**
Nicotine exposure score	71.93 ± 0.49	42.87 ± 1.16	69.23 ± 0.50	91.91 ± 0.43	**< 0.0001**
Sleep duration score	83.90 ± 0.28	66.52 ± 0.71	83.31 ± 0.28	92.99 ± 0.31	**< 0.0001**
BMI score	60.26 ± 0.44	30.74 ± 0.76	55.88 ± 0.41	85.17 ± 0.45	**< 0.0001**
Blood lipid score	64.18 ± 0.36	42.20 ± 0.81	60.67 ± 0.40	83.43 ± 0.52	**< 0.0001**
Blood glucose score	86.59 ± 0.25	61.37 ± 0.74	86.40 ± 0.24	97.92 ± 0.19	**< 0.0001**
Blood pressure score	70.11 ± 0.36	45.5 ± 0.77	66.89 ± 0.39	89.65 ± 0.41	**< 0.0001**

Mean ± standard deviation for continuous variables: *P*-value was calculated by weighted linear regression model. Number (%) for categorical variables: *P*-value was calculated by weighted χ^2^ test. CVH, cardiovascular health; PIR, poverty-to-income ratio; CKD, chronic kidney disease; CVD, cardiovascular disease; ALT, alanine aminotransferase; AST, aspartate aminotransferase; GGT, gamma-glutamyl transferase; ALP, alkaline phosphatase; HEI-2015, Healthy Eating Index-2015; BMI, body mass index. Bold values indicate statistically significant differences among the three CVH groups (*P* < 0.05).

### 3.2 Associations of CVH scores with markers of liver function

In the fully adjusted model, significant associations were observed between CVH scores and all liver function markers (all P for trend < 0.001). Each 1-point increase in CVH score was associated with changes in ALT (β = −0.200 U/L; 95% CI: −0.223, −0.176), AST (β = −0.043 U/L; 95% CI: −0.062, −0.024), GGT (β = −0.453 U/L; 95% CI: −0.509, −0.397), ALP (β = −0.310 U/L; 95% CI: −0.340, −0.281), albumin (β = 0.040 g/dL; 95% CI: 0.0036, 0.045) and AST/ALT ratio (β = 0.0056; 95% CI: 0.0051, 0.0061). Compared with participants with low CVH (0–49), those with high CVH (80–100) demonstrated lower levels of ALT (−7.978 U/L; 95% CI: −9.087, −6.870), AST (−1.571 U/L; 95% CI: −2.428, −0.713), GGT (−16.836 U/L; 95% CI: −19.619, −14.054), and ALP (−12.024 U/L; 95% CI: −13.763, −10.286), and higher albumin (1.617 g/dL; 95% CI: 1.404, 1.830) and AST/ALT ratio (0.229; 95% CI: 0.204, 0.252) (see Table 2).

**TABLE 2:** Associations of CVH scores with markers of liver function.

Markers of liver function	Model	CVH score β (95% CI)	Low (0–49) β (95% CI)	Moderate (50–79) β (95% CI)	High (80–100) β (95% CI)	*P* for trend
ALT	Model 1	−0.158 (−0.178, −0.138), < 0.001	Ref	−1.861 (−2.829, −0.893), < 0.001	−6.349 (−7.430, −5.268), < 0.001	< 0.001
	Model 2	−0.179 (−0.200, −0.158), < 0.001	Ref	−2.946 (−3.877, −2.015), < 0.001	−7.327 (−8.382, −6.271), < 0.001	< 0.001
	Model 3	−0.200 (−0.223, −0.176), < 0.001	Ref	−3.380 (−4.363, −2.398), < 0.001	−7.978 (−9.087, −6.870), < 0.001	< 0.001
AST	Model 1	−0.048 (−0.064, −0.033), < 0.001	Ref	−0.587 (−1.275, 0.100), 0.097	−1.834 (−2.620, −1.049), < 0.001	< 0.001
	Model 2	−0.038 (−0.055, −0.021), < 0.001	Ref	−0.707 (−1.399, −0.015), 0.048	−1.470 (−2.282, −0.658), < 0.001	< 0.001
	Model 3	−0.043 (−0.062, −0.024), < 0.001	Ref	−0.701 (−1.421, 0.019), 0.059	−1.571 (−2.428, −0.713), < 0.001	< 0.001
GGT	Model 1	−0.473 (−0.519, −0.427), < 0.001	Ref	−9.402 (−12.048, −6.755), < 0.001	−19.003 (−21.646, −16.360), < 0.001	< 0.001
	Model 2	−0.454 (−0.506, −0.403), < 0.001	Ref	−9.542 (−12.237, −6.846), < 0.001	−17.871 (−20.585, −15.157), < 0.001	< 0.001
	Model 3	−0.453 (−0.509, −0.397), < 0.001	Ref	−8.775 (−11.578, −5.972), < 0.001	−16.836 (−19.619, −14.054), < 0.001	< 0.001
ALP	Model 1	−0.388 (−0.415, −0.361), < 0.001	Ref	−7.554 (−9.099, −6.009), < 0.001	−16.175 (−17.768, −14.582), < 0.001	< 0.001
	Model 2	−0.348 (−0.376, −0.320), < 0.001	Ref	−6.758 (−8.304, −5.213), < 0.001	−14.171 (−15.780, −12.562), < 0.001	< 0.001
	Model 3	−0.310 (−0.340, −0.281), < 0.001	Ref	−5.503 (−7.172, −3.835), < 0.001	−12.024 (−13.763, −10.286), < 0.001	< 0.001
Albumin	Model 1	0.050 (0.046, 0.055), < 0.001	Ref	1.260 (1.066, 1.454), < 0.001	2.214 (1.979, 2.450), < 0.001	< 0.001
	Model 2	0.042 (0.038, 0.047), < 0.001	Ref	0.928 (0.751, 1.104), < 0.001	1.774 (1.553, 1.994), < 0.001	< 0.001
	Model 3	0.040 (0.036, 0.045), < 0.001	Ref	0.814 (0.641, 0.988), < 0.001	1.617 (1.404, 1.830), < 0.001	< 0.001
AST/ALT	Model 1	0.0039 (0.0034, 0.0044), < 0.001	Ref	0.038 (0.019, 0.057), < 0.001	0.159 (0.135, 0.184), < 0.001	< 0.001
	Model 2	0.0048 (0.0043, 0.0052), < 0.001	Ref	0.069 (0.051, 0.086), < 0.001	0.199 (0.176, 0.221), < 0.001	< 0.001
	Model 3	0.0056 (0.0051, 0.0061), < 0.001	Ref	0.091 (0.073, 0.110), < 0.001	0.229 (0.204, 0.252), < 0.001	< 0.001

Model 1 did not include any covariate. Model 2 was adjusted for age, gender and race/ethnicity. Model 3 was adjusted for age, gender, race/ethnicity, educational level, marital status, PIR, alcohol consumption, history of CVD, CKD, cancer and use of hepatotoxic and hepatoprotective medications. CVH, cardiovascular health; CI, confidence interval; PIR, poverty−income ratio; CVD, cardiovascular disease; CKD, chronic kidney disease.

### 3.3 Restricted cubic spline analysis

**FIGURE 2:**
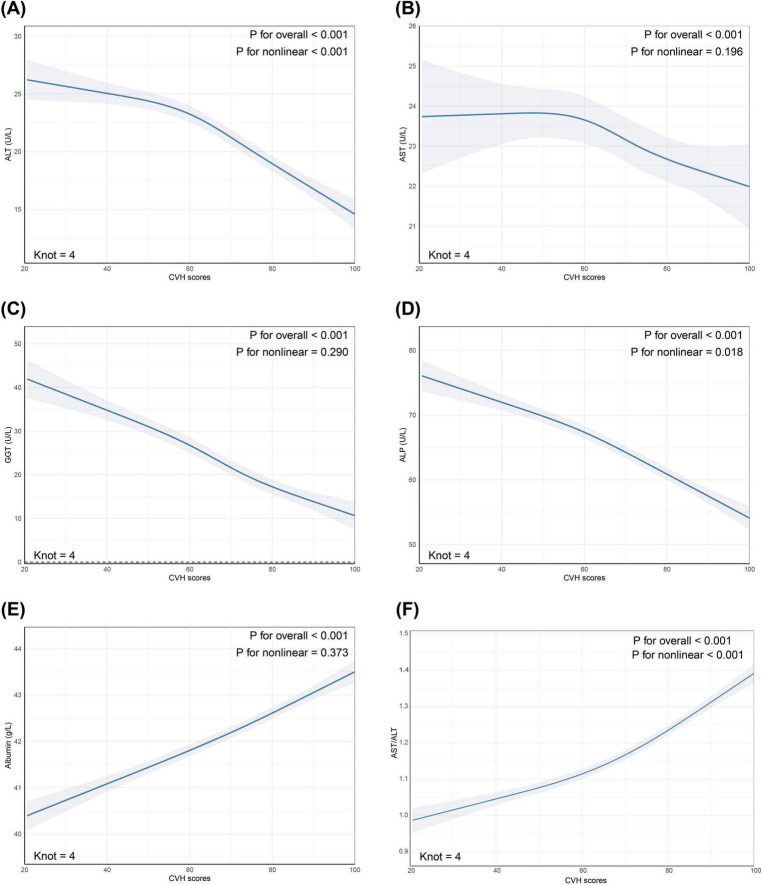
Restricted cubic spline (RCS) analysis revealed non-linear relationships between CVH scores and **(A)** ALT; **(B)** AST; **(C)** GGT; **(D)** ALP; **(E)** Albumin; **(F)** AST/ALT. The markers of liver function (depicted in Steel blue) and 95% CIs (represented by shaded areas) were adjusted for age, sex, race/ethnicity, educational level, marital status, PIR, alcohol consumption, history of CVD, CKD, cancer, and use of hepatotoxic and hepatoprotective medications. CVH, cardiovascular health; ALT, alanine aminotransferase; AST, aspartate aminotransferase; GGT, gamma-glutamyl transferase; ALP, alkaline phosphatase; CI, confidence interval; PIR, poverty-to-income ratio; CVD, cardiovascular disease; CKD, chronic kidney disease.

Restricted cubic spline analyses were performed to examine potential non-linear relationships between CVH scores and liver function markers after full adjustment for confounders. The associations of CVH scores with ALT (P for non-linearity < 0.001), ALP (P for non-linearity = 0.018) and AST/ALT ratio (P for non-linearity < 0.001) demonstrated significant non-linear patterns. In contrast, the relationships between CVH scores and AST (P for non-linearity = 0.196), GGT (P for non-linearity = 0.290), and albumin (P for non-linearity = 0.373) appeared linear (refer to Figure 2).

### 3.4 Subgroup analysis

**FIGURE 3:**
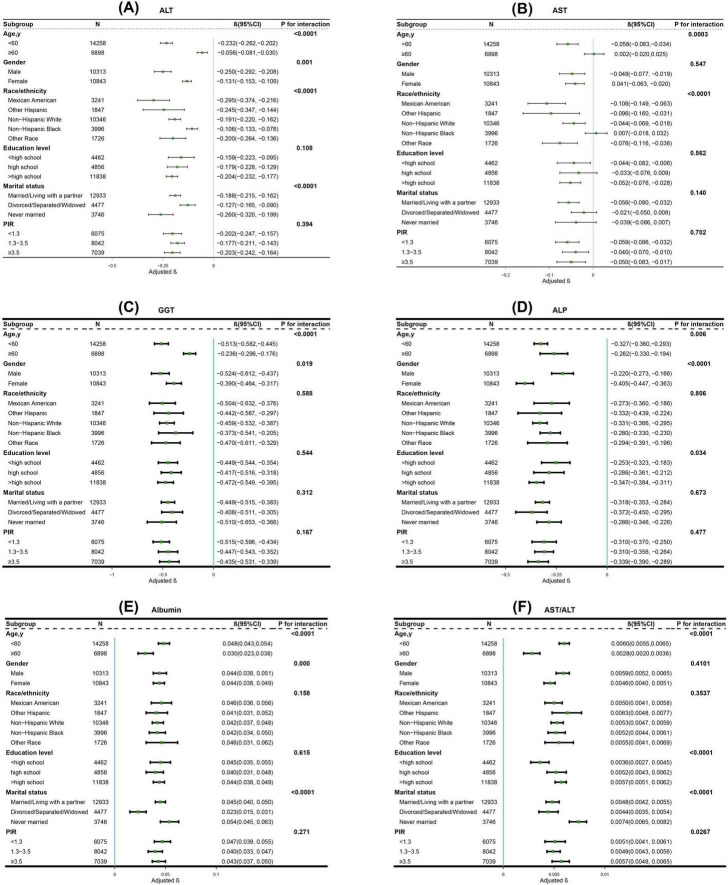
Subgroup analysis for the association between CVH scores and **(A)** ALT; **(B)** AST; **(C)** GGT; **(D)** ALP; **(E)** Albumin; **(F)** AST/ALT. The β coefficients (depicted in green) and 95% CIs (represented by horizontal lines) were adjusted for age, sex, race/ethnicity, educational level, marital status, PIR, alcohol consumption, history of CVD, CKD, cancer, and use of hepatotoxic and hepatoprotective medications, except for the stratification variables themselves. CVH, cardiovascular health; ALT, alanine aminotransferase; AST, aspartate aminotransferase; GGT, gamma-glutamyl transferase; ALP, alkaline phosphatase; CI, confidence interval; PIR, poverty-to-income ratio; CVD, cardiovascular disease; CKD, chronic kidney disease.

Subgroup analyses demonstrated significant interactions between CVH scores and demographic factors, particularly age, in relation to liver function markers. Notably, age exhibited the most robust modification effects on the associations with all liver markers examined, including ALT (P for interaction < 0.0001), AST (P for interaction = 0.0003), GGT (P for interaction < 0.0001), ALP (P for interaction = 0.0064), albumin (P for interaction < 0.0001), and AST/ALT ratio (P for interaction < 0.0001) (Figure 3).

### 3.5 Correlation analyses between CVH components and markers of liver function

**FIGURE 4:**
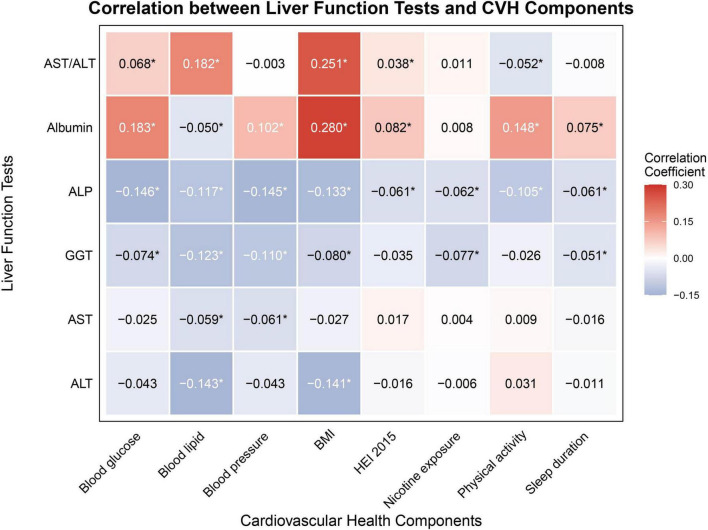
Pearson’s correlation of CVH components and markers of liver function. CVH, cardiovascular health; ALT, alanine aminotransferase; AST, aspartate aminotransferase; GGT, gamma-glutamyl transferase; ALP, alkaline phosphatase; BMI, body mass index; HEI-2015, Healthy Eating Index-2015. *Indicates statistical significance with *P* < 0.05.

Correlation analysis revealed distinct patterns between liver function markers and CVH components. The AST/ALT ratio showed significant positive correlations with BMI (r = 0.251, *P* < 0.05) and blood lipids (r = 0.182, *P* < 0.05). Albumin demonstrated the strongest positive correlations, particularly with BMI (r = 0.280, *P* < 0.05) and blood glucose (r = 0.183, *P* < 0.05). Conversely, ALP and GGT exhibited consistent negative correlations with most CVH components, while ALT and AST showed relatively modest correlations overall (refer to Figure 4) (detailed correlation coefficients are provided in Supplementary Table 2).

### 3.6 Associations of CVH components with markers of liver function in WQS regression and QGC analysis

**FIGURE 5:**
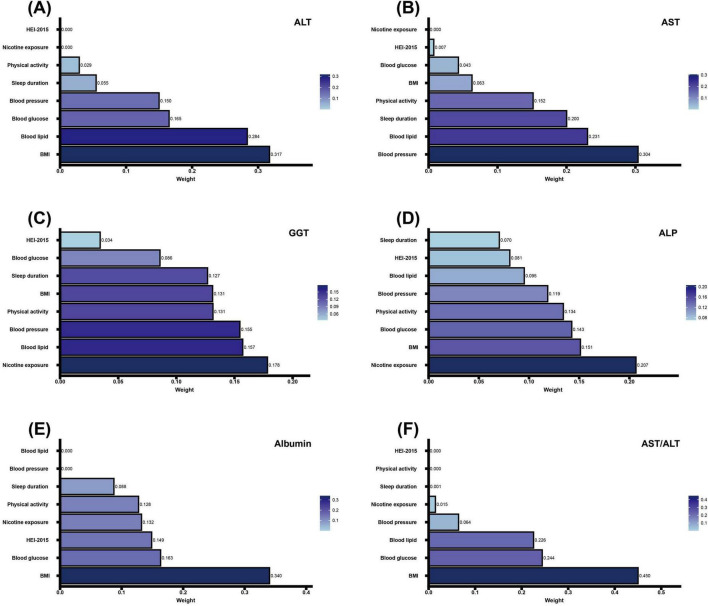
Association of CVH components in WQS regression with markers of liver function. **(A–D)** Negative correlation analysis of CVH components with ALT, AST, GGT, ALP and Albumin in WQS regression. **(E,F)** Positive correlation analysis of CVH components with Albumin and AST/ALT in WQS regression. The model adjusted for age, sex, race/ethnicity, educational level, marital status, PIR, alcohol consumption, history of CVD, CKD, cancer, and use of hepatotoxic and hepatoprotective medications. CVH, cardiovascular health; WQS, weighted quantile sum; ALT, alanine aminotransferase; AST, aspartate aminotransferase; GGT, gamma-glutamyl transferase; ALP, alkaline phosphatase; BMI, body mass index; HEI-2015, Healthy Eating Index-2015; PIR, poverty-to-income ratio; CVD, cardiovascular disease; CKD, chronic kidney disease.

**FIGURE 6:**
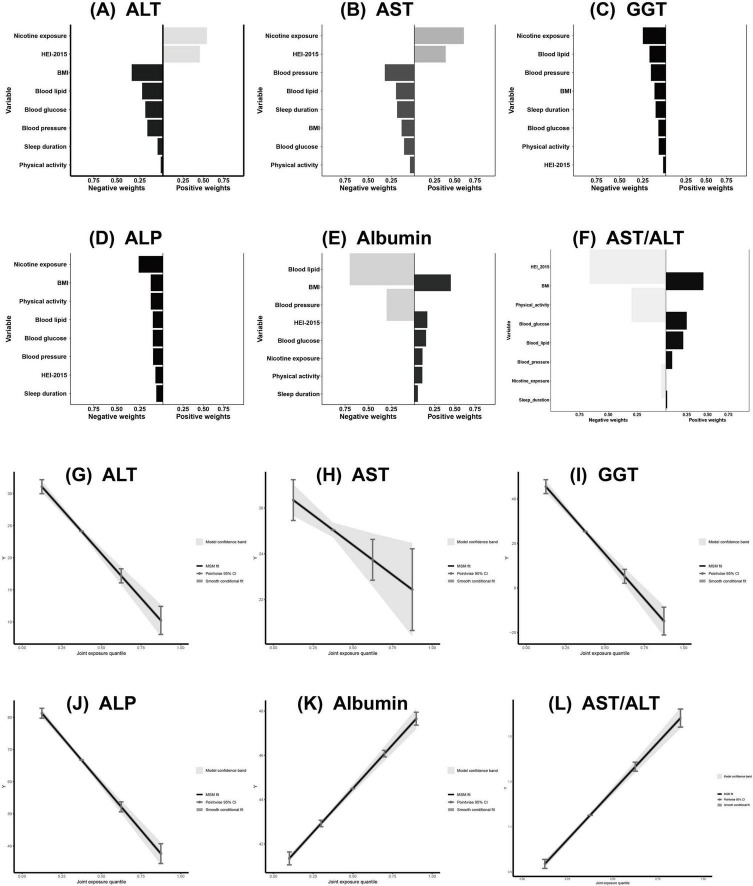
Association of CVH components in QGC analysis with ALT, AST, GGT, ALP, Albumin and AST/ALT **(A–L)**. The model adjusted for age, sex, race/ethnicity, educational level, marital status, PIR, alcohol consumption, history of CVD, CKD, cancer, and use of hepatotoxic and hepatoprotective medications. CVH, cardiovascular health; QGC, quantile G-computation; ALT, alanine aminotransferase; AST, aspartate aminotransferase; GGT, gamma-glutamyl transferase; ALP, alkaline phosphatase; PIR, poverty-to-income ratio; CVD, cardiovascular disease; CKD, chronic kidney disease.

We examined associations between CVH components and liver function markers using both WQS and QGC analyses (Figures 5, 6 and Tables 3, 4).

**TABLE 3:** Association of CVH components with ALT, AST, GGT, ALP, albumin and AST/ALT in WQS regression.

	β coefficient (95% CI)	*P*-value	Direction of WQS
ALT	−8.05 (−8.70, −7.39)	< 0.001	Negative
AST	−2.48 (−3.04, −1.92)	< 0.001	Negative
GGT	−22.17 (−24.88, −19.46)	< 0.001	Negative
ALP	−14.02 (−15.49, −12.55)	< 0.001	Negative
Albumin	2.39 (2.24, 2.53)	< 0.001	Positive
AST/ALT	0.22 (0.21, 0.23)	< 0.001	Positive

The model adjusted for age, sex, race/ethnicity, educational level, marital status, PIR, alcohol consumption, history of CVD, CKD, cancer, and use of hepatotoxic and hepatoprotective medications. CVH, cardiovascular health; ALT, alanine aminotransferase; AST, aspartate aminotransferase; GGT, gamma-glutamyl transferase; ALP, alkaline phosphatase; WQS, weighted quantile sum; CI, confidence interval; PIR, poverty-to-income ratio; CVD, cardiovascular disease; CKD, chronic kidney disease.

**TABLE 4:** Association of CVH components with ALT, AST, GGT, ALP, Albumin and AST/ALT in QGC regression.

	β coefficient (95% CI)	*P*-value
ALT	−6.94 (−8.04, −5.85)	< 0.001
AST	−1.30 (−2.19, −0.41)	0.004
GGT	−20.15 (−23.27, −17.02)	< 0.001
ALP	−14.53 (−16.07, −12.99)	< 0.001
Albumin	1.57 (1.43, 1.72)	< 0.001
AST/ALT	0.22 (0.20, 0.24)	< 0.001

The model adjusted for age, sex, race/ethnicity, educational level, marital status, PIR, alcohol consumption, history of CVD, CKD, cancer, and use of hepatotoxic and hepatoprotective medications. CVH, cardiovascular health; ALT, alanine aminotransferase; AST, aspartate aminotransferase; GGT, gamma-glutamyl transferase; ALP, alkaline phosphatase; QGC, quantile G-computation; CI, confidence interval; PIR, poverty-to-income ratio; CVD, cardiovascular disease; CKD, chronic kidney disease.

Weighted quantile sum analysis revealed significant associations between CVH and liver function markers (all *P* < 0.001). Higher CVH scores were inversely associated with liver enzymes: ALT (β = −8.05; 95% CI: −8.70, −7.39), AST (β = −2.48; 95% CI: −3.04, −1.92), GGT (β = −22.17; 95% CI: −24.88, −19.46), and ALP (β = −14.02; 95% CI: −15.49, −12.55), and positively associated with albumin (β = 2.39; 95% CI: 2.24, 2.53) and AST/ALT ratio (β = 0.22; 95% CI: 0.21, 0.23) (Table 3).

Component-specific analysis identified the primary contributors for each marker: BMI and blood lipids for ALT (Figure 5A); blood pressure and blood lipids for AST (Figure 5B); nicotine exposure and blood lipids for GGT (Figure 5C); nicotine exposure and BMI for ALP (Figure 5D); BMI and blood glucose for albumin and AST/ALT ratio (Figures 5E, F).

quantile g-computation analysis similarly showed significant associations between CVH and liver function markers (all *P* < 0.05). Higher CVH scores were inversely associated with liver enzymes: ALT (β = −6.94; 95% CI: −8.04, −5.85), AST (β = −1.30; 95% CI: −2.19, −0.41), GGT (β = −20.15; 95% CI: −23.27, −17.02), and ALP (β = −14.53; 95% CI: −16.07, −12.99), and positively associated with albumin (β = 1.57; 95% CI: 1.43, 1.72) and AST/ALT ratio (β = 0.22; 95% CI: 0.20, 0.24) (Table 4). Component-specific analysis identified nicotine exposure and diet quality as primary contributors to ALT and AST variations (Figures 6A, B); nicotine exposure and blood lipids to GGT (Figure 6C); nicotine exposure and BMI to ALP (Figure 6D); blood lipids and BMI to albumin levels (Figure 6E) and diet quality and BMI to AST/ALT ratio (Figure 6F). Joint exposure quartile analysis demonstrated consistent dose-response relationships between CVH and all liver function markers (Figures 6G–L).

### 3.7 Sensitivity analysis

Sensitivity analyses showed minimal differences in age (standardized difference = 0.06) and sex (0.00) between included and excluded participants. The main differences were observed in race/ethnicity (0.28), education level (0.22), and poverty-income ratio (0.17), while clinical characteristics showed small differences (CKD = 0.07, CVD = 0.05, cancer = 0.01) (detailed in Supplementary Table 3). Second, we generated E-values to assess the sensitivity to unmeasured confounding. The primary findings were robust, unless unmeasured confounders existed with associations of 2.52 (GGT), 2.07 (ALP), and 1.74 (ALT) for both the exposure and outcome (refer to Supplementary Table 4).

## 4 Discussion

Using NHANES data from a representative sample of 21,156 United States adults, we examined associations between CVH, measured by LE8, and liver function markers. Multivariate analyses revealed that higher CVH scores were significantly associated with improved liver function profiles. Each 1-point increase in CVH score corresponded to decreased liver enzyme levels (ALT: β = −0.200 U/L, 95% CI: −0.223, −0.176; AST: β = −0.043 U/L, 95% CI: −0.062, −0.024; GGT: β = −0.453 U/L, 95% CI: −0.509, −0.397; ALP: β = −0.310 U/L, 95% CI: −0.340, −0.281) and increased albumin concentration (β = 0.040 g/dL, 95% CI: 0.036, 0.045) and AST/ALT ratio (β + 0.0056, 95%CI: 0.0051, 0.0061).

Restricted cubic spline analyses revealed complex non-linear relationships between cardiovascular health and certain liver enzymes. Notably, we observed significant non-linear associations for ALT (P for non-linearity < 0.001), ALP (P for non-linearity = 0.018) and AST/ALT ratio (P for non-linearity < 0.001), while relationships with AST, GGT, and albumin maintained linearity. The non-linear patterns demonstrated an accelerated improvement in both ALT, ALP and AST/ALT ratio levels as cardiovascular health scores increased, suggesting potential threshold effects or synergistic benefits of achieving multiple ideal cardiovascular health metrics simultaneously. These enhanced effects at higher CVH scores may be attributed to several well-established physiological mechanisms, including improved endothelial function leading to better vasodilator production and reduced inflammatory responses (30), and enhanced hepatic blood flow optimizing liver sinusoidal endothelial cell function and metabolic regulation (31, 32).

Furthermore, our subgroup analyses uncovered significant age-related differences in these associations (P for interaction < 0.0001 for ALT, AST, GGT, albumin and AST/ALT ratio; P for interaction = 0.0064 for ALP). Particularly, individuals younger than 60 years demonstrated more pronounced improvements in liver function markers with increasing cardiovascular health scores compared to their older counterparts. Several mechanisms might explain this age-specific pattern. First, younger individuals typically possess greater physiological plasticity and regenerative capacity, enabling their liver to respond more robustly to cardiovascular health improvements (33). Second, their shorter cumulative exposure to risk factors likely results in less established pathological changes, potentially making them more responsive to positive cardiovascular health modifications (34). Third, age-related changes in hepatic blood flow, metabolic capacity, and inflammatory responses could attenuate the beneficial effects of cardiovascular health improvements in older adults (35).

To evaluate the complex relationships between CVH components and liver function, we employed complementary analytical approaches: WQS regression and QGC analyses. These methods revealed distinct component-specific contributions due to their fundamental methodological differences. WQS regression, utilizing unidirectional associations with constrained non-negative weights, identified BMI and blood lipids as principal contributors to ALT levels, while QGC analysis, incorporating bidirectional associations, highlighted nicotine exposure and diet quality as key factors. For albumin, WQS regression emphasized BMI and blood glucose, whereas QGC analysis identified lipids and BMI as primary determinants. Despite methodological variations, both approaches consistently demonstrated robust CVH-liver function associations.

The observed relationships are supported by established pathophysiological mechanisms. The paradoxical AST/ALT ratio elevation in high CVH groups reflects metabolic optimization rather than hepatic pathology. ALT’s predominant decrease (−23.0% vs. AST −7.1%) indicates improved hepatocyte metabolic efficiency, contrasting with the AST elevation seen in mitochondrial damage during fibrosis progression. BMI elevation promotes insulin resistance and lipid metabolism disorders, increasing NAFLD risk, while altering serum amino acid profiles—particularly elevated branched-chain amino acids and glutamate—potentially exacerbating hepatic steatosis and inflammation (36). Glucose metabolism abnormalities, particularly diabetes, enhance liver fibrosis risk through oxidative stress pathway activation and chronic inflammatory response promotion, while disrupting hepatic gluconeogenesis and insulin metabolism (37). Dyslipidemia manifests hepatic dysfunction in lipid metabolism through elevated low-density lipoprotein cholesterol and triglycerides, with decreased HDL-c, potentially intensifying hepatocellular injury via lipotoxicity (38). Dietary quality exerts complex effects: high-fat, high-sugar diets may induce liver inflammation and fibrosis by altering gut microbiota composition, increasing intestinal permeability, and promoting endotoxemia. Conversely, plant-based diets, particularly the Mediterranean diet, may protect liver health by promoting beneficial bacterial growth and enhancing intestinal barrier function (39, 40). Sleep deficiency impacts liver health through multiple pathways: sympathetic nervous system activation, altered appetite-regulating hormone levels, and increased oxidative stress and pro-inflammatory factor expression, collectively inducing hepatic lipid metabolism disorders and enhanced inflammatory responses (41). Regular physical activity confers hepatoprotective effects by enhancing insulin sensitivity, promoting fatty acid oxidation, and reducing systemic inflammation and oxidative stress. Higher physical activity levels demonstrate significant associations with reduced liver fibrosis risk (42). Tobacco exposure compromises liver function through dual mechanisms: direct cytotoxic effects and indirect metabolic disruption. These processes increase pro-inflammatory cytokine production—including interleukin-1 (IL-1), interleukin-6 (IL-6), and tumor necrosis factor-alpha (TNF-α)—while disrupting cholesterol and bile acid metabolism homeostasis (36, 43).

This study represents the first comprehensive investigation of CVH’s relationship with liver function using the novel Life’s Essential 8 metric. Our integrated analytical approach uniquely revealed differential contributions of CVH components and identified previously unrecognized non-linear relationships for ALT, ALP and AST/ALT ratio. These findings extend beyond previous research focused on individual cardiovascular components, providing a more nuanced understanding of CVH-liver function interactions.

Several methodological limitations need to be considered. First, the cross-sectional design precludes causal inference, while single time-point measurements may inadequately capture temporal variations in both CVH and liver function markers, necessitating longitudinal studies to establish temporal relationships. Second, though E-value sensitivity analysis suggested robust associations, the NHANES database lacks screening for autoimmune liver diseases and nephrotic syndrome, particularly those associated with elderly women, which might affect the interpretation of our findings. Third, the exclusion of participants with incomplete LE8 data or unclear medical histories may have introduced selection bias and reduced population representativeness, although our sensitivity analyses suggested that differences between included and excluded participants were primarily in socioeconomic factors rather than clinical characteristics. Fourth, while the exclusion of participants with viral hepatitis (HBV/HCV infections) was necessary to minimize confounding effects on liver function parameters and strengthen internal validity, this approach limits the generalizability of our findings to populations with viral hepatitis, suggesting the need for dedicated studies in these populations. Fifth, while AST/ALT ratio provides valuable insights into liver function, its limited sensitivity for early-stage fibrosis should be noted. Future studies incorporating more advanced diagnostic techniques, such as transient elastography or other direct fibrosis markers, are warranted to better characterize the relationship between CVH and liver fibrosis progression.

## 5 Conclusion

In summary, our study demonstrated significant associations between cardiovascular health, assessed through LE8, and liver function markers in United States adults. Through complementary analytical approaches, WQS and QGC analyses identified nicotine exposure, BMI and blood lipids as primary contributors to these relationships. RCS analysis revealed novel non-linear associations for ALT ALP and AST/ALT ratio. Our findings provide novel evidence for the intricate relationship between cardiovascular and liver health using this comprehensive CVH assessment tool, suggesting potential targets for integrated cardiovascular-hepatic health interventions.

## Data Availability

Publicly available datasets were analyzed in this study. This data can be found here: the datasets analyzed in this study are publicly available from the National Health and Nutrition Examination Survey (NHANES), maintained by the National Center for Health Statistics (NCHS). The data can be accessed directly at the NHANES website: https://www.cdc.gov/nchs/nhanes/index.htm.

## Footnotes

1
http://www.empowerstats.net/en/
