# Supplementary material for: Association between cardiovascular health and markers of liver function: a cross-sectional study from NHANES 2005–2018
Source: Front Med (Lausanne). 2025 Mar 12;12:1538654. doi: 10.3389/fmed.2025.1538654 (PMC11936933; doi:10.3389/fmed.2025.1538654)
Supplement: Supplementary file 2 [file Table_2.docx]

**Supplementary Table 2. Correlation Analysis between Markers of Liver Function and Cardiovascular Health (CVH) components.**

| **Markers of Liver Function** | **CVH components** | **Correlation Coefficient (95% CI)** |
| --- | --- | --- |
| **ALT** | HEI-2015 | -0.016 (-0.029, -0.002)* |
|  | Physical activity | 0.031 (0.018, 0.045)* |
|  | Nicotine exposure | -0.006 (-0.019, 0.008) |
|  | Sleep duration | -0.011 (-0.024, 0.003) |
|  | BMI | -0.141 (-0.155, -0.128)* |
|  | Blood lipid | -0.143 (-0.156, -0.129)* |
|  | Blood glucose | -0.043 (-0.056, -0.029)* |
|  | Blood pressure | -0.043 (-0.056, -0.029)* |
| **AST** | HEI-2015 | 0.017 (0.004, 0.031)* |
|  | Physical activity | 0.009 (-0.004, 0.023) |
|  | Nicotine exposure | 0.004 (-0.009, 0.018) |
|  | Sleep duration | -0.016 (-0.029, -0.002)* |
|  | BMI | -0.027 (-0.041, -0.014)* |
|  | Blood lipid | -0.059 (-0.073, -0.046)* |
|  | Blood glucose | -0.025 (-0.039, -0.012)* |
|  | Blood pressure | -0.061 (-0.074, -0.047)* |
| **GGT** | HEI-2015 | -0.035 (-0.048, -0.021)* |
|  | Physical activity | -0.026 (-0.039, -0.012)* |
|  | Nicotine exposure | -0.077 (-0.091, -0.064)* |
|  | Sleep duration | -0.051 (-0.065, -0.038)* |
|  | BMI | -0.080 (-0.094, -0.067)* |
|  | Blood lipid | -0.123 (-0.136, -0.110)* |
|  | Blood glucose | -0.074 (-0.087, -0.060)* |
|  | Blood pressure | -0.110 (-0.123, -0.097)* |
| **ALP** | HEI-2015 | -0.061 (-0.074, -0.047)* |
|  | Physical activity | -0.105 (-0.118, -0.092)* |
|  | Nicotine exposure | -0.062 (-0.075, -0.049)* |
|  | Sleep duration | -0.061 (-0.075, -0.048)* |
|  | BMI | -0.133 (-0.147, -0.120)* |
|  | Blood lipid | -0.117 (-0.130, -0.104)* |
|  | Blood glucose | -0.146 (-0.159, -0.133)* |
|  | Blood pressure | -0.145 (-0.158, -0.132)* |
| **Albumin** | HEI-2015 | 0.082 (0.068, 0.095)* |
|  | Physical activity | 0.148 (0.135, 0.161)* |
|  | Nicotine exposure | 0.008 (-0.005, 0.022) |
|  | Sleep duration | 0.075 (0.061, 0.088)* |
|  | BMI | 0.280 (0.267, 0.292)* |
|  | Blood lipid | -0.050 (-0.063, -0.036)* |
|  | Blood glucose | 0.183 (0.170, 0.196)* |
|  | Blood pressure | - 1. (0.089, 0.116)* |
| AST/ALT | HEI-2015 | 0.038 (0.024, 0.051)* |
|  | Physical activity | -0.052 (-0.065, -0.038)* |
|  | Nicotine exposure | 0.011 (-0.002, 0.025) |
|  | Sleep duration | -0.008 (-0.022, 0.005) |
|  | BMI | 0.251 (0.238, 0.263)* |
|  | Blood lipid | 0.182 (0.169, 0.195)* |
|  | Blood glucose | 0.068 (0.055, 0.081)* |
|  | Blood pressure | -0.003 (-0.017, 0.010) |

Notes:

1. All correlation coefficients were calculated using Pearson's correlation test.

2. *: P < 0.05 was considered statistically significant.

**Abbreviations**: CVH: cardiovascular health; CI: confidence interval; ALT: Alanine Aminotransferase; AST: Aspartate Aminotransferase; GGT: Gamma-Glutamyl Transferase; ALP: Alkaline Phosphatase; BMI: Body Mass Index; HEI-2015: Healthy Eating Index-2015.
