# Supplementary material for: Association between cardiovascular health and markers of liver function: a cross-sectional study from NHANES 2005–2018
Source: Front Med (Lausanne). 2025 Mar 12;12:1538654. doi: 10.3389/fmed.2025.1538654 (PMC11936933; doi:10.3389/fmed.2025.1538654)
Supplement: Supplementary file 3 [file Table_3.docx]

**Supplementary Table 3. Comparison of Baseline Characteristics Between Included and Excluded Participants**

| **Characteristics** | **Included** | **Excluded** | **Standardized difference** | **P-value** |
| --- | --- | --- | --- | --- |
| N | 21156 | 14817 |  |  |
| Age, y | 49.15 ± 17.72 | 50.18 ± 18.58 | 0.06 (0.04, 0.08) | <0.001 |
| Sex, % |  |  | 0.00 (-0.02, 0.02) | 0.831 |
| Male | 48.75 | 48.63 |  |  |
| Female | 51.25 | 51.37 |  |  |
| Race/ethnicity, % |  |  | 0.28 (0.26, 0.30) | <0.001 |
| Mexican American | 15.32 | 17.45 |  |  |
| Other Hispanic | 8.73 | 10.89 |  |  |
| Non-Hispanic White | 48.90 | 35.86 |  |  |
| Non-Hispanic Black | 18.89 | 22.99 |  |  |
| Other Race | 8.16 | 12.82 |  |  |
| Education level, % |  |  | 0.22 (0.20, 0.24) | <0.001 |
| ＜high school | 21.09 | 30.13 |  |  |
| High school | 22.95 | 22.83 |  |  |
| ＞high school | 55.96 | 47.04 |  |  |
| Marital status, % |  |  | 0.11 (0.09, 0.13) | <0.001 |
| Married/Living with a partner | 61.13 | 55.74 |  |  |
| Divorced/Separated/Widowed | 21.16 | 24.34 |  |  |
| Never married | 17.71 | 19.91 |  |  |
| PIR, % |  |  | 0.17 (0.15, 0.20) | <0.001 |
| <1.3 | 28.72 | 35.77 |  |  |
| 1.3-3.5 | 38.01 | 37.60 |  |  |
| ≥3.5 | 33.27 | 26.62 |  |  |
| Alcohol consumption, % |  |  | 0.09 (0.07, 0.12) | <0.001 |
| Yes | 71.90 | 67.68 |  |  |
| No | 28.10 | 32.32 |  |  |
| CKD |  |  | 0.07 (0.05, 0.10) | <0.001 |
| Yes | 17.07 | 19.92 |  |  |
| No | 82.93 | 80.08 |  |  |
| Cancer, % |  |  | 0.01 (-0.01, 0.03) | 0.497 |
| Yes | 9.91 | 9.69 |  |  |
| No | 90.09 | 90.31 |  |  |
| CVD |  |  | 0.05 (0.03, 0.07) | <0.001 |
| Yes | 10.47 | 12.18 |  |  |
| No | 89.53 | 87.82 |  |  |
| Hepatotoxic medications, % |  |  | 0.02 (-0.00, 0.04) | 0.091 |
| Yes | 78.03 | 78.78 |  |  |
| No | 21.97 | 21.22 |  |  |
| Hepatoprotective medications, % |  |  | 0.01 (-0.01, 0.03) | 0.196 |
| Yes | 98.51 | 98.67 |  |  |
| No | 1.49 | 1.33 |  |  |

**Note:** Data are presented as mean ± SD for continuous variables and percentages for categorical variables. Standardized differences are presented with 95% confidence intervals in parentheses.

**Abbreviations:** PIR, poverty-to-income ratio; CKD, chronic kidney disease; CVD, cardiovascular disease.
