# Supplementary material for: Association between cardiovascular health and markers of liver function: a cross-sectional study from NHANES 2005–2018
Source: Front Med (Lausanne). 2025 Mar 12;12:1538654. doi: 10.3389/fmed.2025.1538654 (PMC11936933; doi:10.3389/fmed.2025.1538654)
Supplement: Supplementary file 4 [file Table_4.docx]

**Supplementary Table 4. Sensitivity Analysis Using E-values for the Associations Between CVH Score and Liver Function Markers in Model 3.**

| **Liver Function Marker** | **β (95% CI)** | **E-value** | **E-value for CI limit** |
| --- | --- | --- | --- |
| ALT | -0.200 (-0.223, -0.176) | 1.74 | 1.67 |
| AST | -0.043 (-0.062, -0.024) | 1.26 | 1.18 |
| GGT | -0.453 (-0.509, -0.397) | 2.52 | 2.34 |
| ALP | -0.310 (-0.340, -0.281) | 2.07 | 1.98 |
| Albumin | 0.040 (0.036, 0.045) | 1.25 | 1.23 |
| AST/ALT ratio | 0.0056 (0.0051, 0.0061) | 1.081 | 1.077 |

**Notes:**

1. Model 3 was adjusted for age, gender, race/ethnicity, educational level, marital status, PIR, alcohol consumption, history of CVD, CKD, cancer and use of hepatotoxic and hepatoprotective medications.
2. E-value represents the minimum strength of association that an unmeasured confounder would need to have with both the exposure (CVH score) and the outcome (liver function marker) to fully explain away the observed association.
3. E-value for CI limit represents the minimum strength of association that an unmeasured confounder would need to have to shift the confidence interval to include the null value.
4. Higher E-values indicate that stronger unmeasured confounding would be needed to explain away the observed associations.
